# Mast cell activation test in the diagnosis of allergic disease and anaphylaxis

**DOI:** 10.1016/j.jaci.2018.01.043

**Published:** 2018-08

**Authors:** Rajia Bahri, Adnan Custovic, Peter Korosec, Marina Tsoumani, Martin Barron, Jiakai Wu, Rebekah Sayers, Alf Weimann, Monica Ruiz-Garcia, Nandinee Patel, Abigail Robb, Mohamed H. Shamji, Sara Fontanella, Mira Silar, E.N.Clare Mills, Angela Simpson, Paul J. Turner, Silvia Bulfone-Paus

**Affiliations:** a Division of Musculoskeletal and Dermatological Sciences & Manchester Collaborative Centre for Inflammation Research (MCCIR), School of Biological Sciences, University of Manchester, Manchester, United Kingdom; b Section of Paediatrics, Department of Medicine, Imperial College London, London, United Kingdom; f Section of Allergy and Clinical Immunology, National Heart and Lung Institute, Imperial College London, London, United Kingdom; c Laboratory for Clinical Immunology & Molecular Genetics, University Hospital for Respiratory and Allergic Diseases, Golnik, Slovenia; d Division of Infection, Immunity and Respiratory Medicine, School of Biological Sciences, University of Manchester, and NIHR Manchester Biomedical Research Centre, Central Manchester University Hospitals NHS Foundation Trust, Manchester Academic Health Science Centre, Manchester, United Kingdom; e EUROIMMUN AG, Lübeck, Germany

**Keywords:** Anaphylaxis, basophil activation test, diagnosis, food allergy, mast cells, mast cell activation test, peanut allergy, AUC, Area under the curve, BAT, Basophil activation test, CD-sens, Threshold sensitivity, CRTH2, Chemoattractant receptor–homologous molecule expressed of T_H_2 cells, DBPCFC, Double-blind, placebo-controlled food challenge, FDA, Functional data analysis, FPC, Functional principal component, hMC, Human blood-derived mast cell, ICC, Intraclass correlation, MAT, Mast cell activation test, MC, Mast cell, PBST, PBS containing 0.1% Tween 20, PGD_2_, Prostaglandin D_2_, ROC, Receiver operating characteristic, sIgE, Allergen-specific IgE, SPT, Skin prick test

## Abstract

**Background:**

Food allergy is an increasing public health issue and the most common cause of life-threatening anaphylactic reactions. Conventional allergy tests assess for the presence of allergen-specific IgE, significantly overestimating the rate of true clinical allergy and resulting in overdiagnosis and adverse effect on health-related quality of life.

**Objective:**

To undertake initial validation and assessment of a novel diagnostic tool, we used the mast cell activation test (MAT).

**Methods:**

Primary human blood-derived mast cells (MCs) were generated from peripheral blood precursors, sensitized with patients' sera, and then incubated with allergen. MC degranulation was assessed by means of flow cytometry and mediator release. We compared the diagnostic performance of MATs with that of existing diagnostic tools to assess in a cohort of peanut-sensitized subjects undergoing double-blind, placebo-controlled challenge.

**Results:**

Human blood-derived MCs sensitized with sera from patients with peanut, grass pollen, and Hymenoptera (wasp venom) allergy demonstrated allergen-specific and dose-dependent degranulation, as determined based on both expression of surface activation markers (CD63 and CD107a) and functional assays (prostaglandin D_2_ and β-hexosaminidase release). In this cohort of peanut-sensitized subjects, the MAT was found to have superior discrimination performance compared with other testing modalities, including component-resolved diagnostics and basophil activation tests. Using functional principle component analysis, we identified 5 clusters or patterns of reactivity in the resulting dose-response curves, which at preliminary analysis corresponded to the reaction phenotypes seen at challenge.

**Conclusion:**

The MAT is a robust tool that can confer superior diagnostic performance compared with existing allergy diagnostics and might be useful to explore differences in effector cell function between basophils and MCs during allergic reactions.

## Graphical abstract

**Figure:**
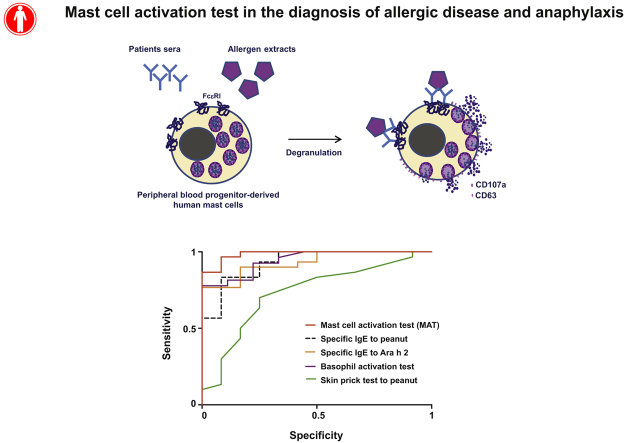

IgE-mediated food allergy is an increasing public health issue with a prevalence of 6% in children and up to 2% in adults.^1^ It is the most common cause of anaphylaxis, a potentially life-threatening and rapidly progressing systemic allergic reaction that can lead to death caused by airway obstruction or cardiovascular collapse.^2^ The adverse effect of food allergy on the quality of life of children and their families is greater than that caused by diabetes and other chronic illnesses.^3^

The gold standard test for diagnosis of food allergy is a double-blind, placebo-controlled food challenge (DBPCFC), in which increasing doses of food (or placebo) are administered under medical supervision.^4^ Open and unblinded oral food challenges are often performed as an alternative. However, oral food challenges are time-consuming, costly, and not without risk because of the potential for anaphylaxis or even death.^5^ In practice, IgE-mediated food allergy is usually diagnosed by using a surrogate marker, detection of allergen-specific IgE (sIgE) to the implicated food (referred to as sensitization) either in serum or through skin prick tests (SPTs). However, sensitization frequently fails to correlate with clinical reactivity: a positive allergy test result (either SPTs or IgE measurement to the whole allergen extract) is not diagnostic in isolation.^6^ A false-positive rate of greater 50% has been reported in population-based studies,^7^, ^8^, ^9^ and consequently, overdiagnosis of food allergy is common.^8^ This results in unnecessary dietary exclusions, social restrictions, and anxiety, which can further impair nutrition and quality of life.^3^

To date, attempts to develop more accurate tests to diagnose food allergy have focused on 2 strategies: component-resolved diagnostics^10^ and the basophil activation test (BAT).^11^ Component-resolved diagnostics use purified native or recombinant allergens to detect sIgE to individual allergenic molecules rather than whole allergen extracts.^10^ Superior diagnostic accuracy has been demonstrated for peanut allergy,^8^ but data are limited and equivocal for other allergens.^12^, ^13^

In the BAT basophils from patients are incubated with allergen *ex vivo*, and surface expression of activation markers is measured by using flow cytometry.^14^ The BAT can improve diagnostic accuracy in patients with peanut allergy^15^ but is technically challenging and limited to a few specialist centers and lacks the accuracy and reproducibility of a food challenge.^15^, ^16^, ^17^ It has not been validated for other food allergens and needs to be evaluated further in terms of feasibility and cost-effectiveness outside specialist units.^11^

Whether basophils are involved as effector cells in the pathophysiology of allergic reactions is unclear.^18^ Traditionally, mast cells (MCs) have been considered the main effector cells in patients with allergic reactions.^18^ After allergen exposure, these cells become activated through IgE cross-linking of FcεRI expressed on the cell surface, resulting in release and *de novo* synthesis of inflammatory mediators.^18^ Despite sharing allergen-mediated activation mechanisms, MCs are transcriptionally distinct and independent from circulating granulocytes.^19^, ^20^ Therefore we sought to develop an alternative approach to the diagnosis of allergic disease and anaphylaxis using primary human blood-derived mast cells (hMCs) generated from CD117^+^ peripheral blood precursors, which are passively sensitized with patients' sera and then incubated *in vitro* with allergen; this is known as the mast cell activation test (MAT). In this report we describe development of the MAT, its potential application in patients with peanut and insect venom allergy, and initial validation as a diagnostic tool for peanut allergy compared with existing diagnostic tests.

## Methods

### Study design

We developed a novel diagnostic tool, the MAT, in which primary hMCs generated from peripheral blood precursors from healthy donors were sensitized passively with patients' sera and then incubated with allergen *in vitro*, and MC activation was assessed. All study participants provided written informed consent (UK NHS Human Research Authority reference 15/NW/040, 15/LO/0286, and 15/LO/0287 and Slovenian National Medical Ethics Committee reference 75/06/15).

### Development of the MAT

#### Generation of hMCs from peripheral blood precursors

hMCs were generated, as previously described.^21^, ^22^, ^23^ Briefly, CD117^+^CD34^+^ cells were purified from buffy coat blood mononuclear cells by using a positive selection kit (Miltenyi Biotec, Bergisch Gladbach, Germany). Cells were cultured in serum-free StemSpan medium (STEMCELL Technologies, Vancouver, British Columbia, Canada) supplemented with 100 U/mL penicillin (Invitrogen, Carlsbad, Calif), 100 μg/mL streptomycin (Invitrogen), human IL-6 (50 ng/mL; PeproTech, Rocky Hill, NJ), human IL-3 (10 ng/mL; PeproTech), human stem cell factor (100 ng/mL; PeproTech), and 10 μg/mL human low-density lipoprotein (STEMCELL Technologies). After 30 days, the cells were transferred progressively to culture medium containing Iscove modified Dulbecco medium with GlutaMAX-I, 50 μmol/L β_2_-mercaptoethanol, 0.5% BSA, 1% Insulin-Transferrin-Selenium (Life Technologies, Grand Island, NY), 100 U/mL penicillin, 100 μg/mL streptomycin, human IL-6 (50 ng/mL), and human stem cell factor (100 ng/mL). After 8 to 10 weeks of culture, the cells were tested for maturity and found to be greater than 90% CD117^+^ and FcεRIa^+^ cells.

We used immunocytochemistry to characterize hMCs generated from peripheral blood precursors (details are provided in the Methods section in this article's Online Repository at www.jacionline.org).

#### Passive sensitization of cultured primary hMCs

Cultured primary hMCs were sensitized passively with serum samples from subjects with a physician-confirmed peanut allergy recruited from the Allergy Centre at the University Hospital of South Manchester. All patients had a convincing history of immediate reaction on exposure to peanut and detectable serum specific IgE to whole peanut extract. Control serum was collected from patients with pollen allergy but no history of peanut allergy who were consuming peanuts and had negative IgE and/or SPT results to whole peanut extract.

To assess whether the MAT could be applied to nonfood allergens, we recruited 28 patients presenting with an acute episode of anaphylaxis to the emergency department of the University Hospital Golnik, Slovenia, caused by an insect sting; 21 patients had a confirmed systemic reaction and sIgE levels to wasp venom, and 7 patients had a confirmed systemic reaction and sIgE levels to honeybee but not wasp venom.

#### MC activation assay

hMCs were cultured in supplemented medium and sensitized passively by means of overnight incubation with the participants' sera (diluted 1:10). Cells were washed and treated with peanut extract at 0.01, 0.1, 1, 10, 100, and 1000 ng/mL protein or 10 nmol/L recombinant peanut allergens rAra h 1, rAra h 2, rAra h 3, rAra h 6, and rAra h 8 or left untreated. Allergen sources are described in detail in the Methods section in this article's Online Repository. As a positive control, sera-sensitized hMCs were incubated with goat anti-human IgE (10 μg/mL; KPL, Gaithersburg, Md). After a 1-hour incubation, hMCs were stained with CD117 (clone 104D2; eBioscience, San Diego, Calif), FcεRIa (clone AER-37; BioLegend, San Diego, Calif), CD63 (clone H5C6; BioLegend), and CD107a (clone H4A3; BD PharMingen, San Jose, Calif) antibodies and analyzed by means of flow cytometry with the LSR II or Fortessa (BD Biosciences) and FlowJo software (FlowJo 7.6.5 and FlowJo-V10; TreeStar, Ashland, Ore). Intracellular tryptase levels were evaluated with an appropriate kit (eBioscience) with anti-human tryptase (clone G3 from EMD Millipore, Billerica, Mass) and a secondary anti-mouse IgG (Poly4053; BioLegend).

To ensure quality control across batches of hMCs, in each run we included a reference positive control and anti-IgE. Each batch was generated from 3 to 9 pooled donors to reduce the risk of specific donor dependence.

#### Measurement of MC mediator secretion

After incubation with allergen, 50-μL aliquots from cell cultures were taken and centrifuged to separate the supernatant and cell pellet. Cell pellets were lysed in 50 μL of media culture 1% Triton X-100. β-Hexosaminidase levels were measured in supernatants, as well as in cell pellets, by adding 100 μL of β-hexosaminidase substrate and 1 mmol/L *p*-nitrophenyl N-acetyl-beta-D-glucosamine (Sigma-Aldrich, St Louis, Mo) in 0.05 mol/L citrate buffer (pH 4.5) for 2 hours at 37°C in a 5% CO_2_ atmosphere. The reaction was stopped by adding 300 μL of 0.05 mol/L sodium carbonate buffer (pH 10). OD was measured at 405 nm. hMC degranulation was assessed as percentage release of total β-hexosaminidase. Prostaglandin D_2_ (PGD_2_) levels were measured in supernatants by using the ELISA kit from Cayman Chemical (Ann Arbor, Mich).

### Validation, diagnostic performance, and comparison of the MAT with other diagnostic tests

#### Study participants, data sources, and other diagnostic tests for peanut allergy

We recruited 42 peanut-sensitized subjects who underwent DBPCFCs to peanut (details are provided in the Methods section in this article's Online Repository). Patients who reacted on DBPCFC were considered to be allergic to peanut, whereas those who passed the challenge without experiencing dose-limiting symptoms were classified as sensitized but peanut tolerant.

Blood samples were collected immediately before challenge and transferred without delay for assessment of basophil activation or centrifuged, and sera were stored at −80°C until analysis.

#### Specific IgE to whole allergen extract, component-resolved diagnostics, and SPTs

Levels of total IgE, peanut-specific IgE, and IgE to the recombinant allergen components rAra h 1, 2, 3, 6, 8, and 9 were measured by using ImmunoCAP (Thermo Fisher Scientific, Uppsala, Sweden). SPTs were undertaken according to national guidelines by using lancets (ALK-Abelló, Hørsholm, Denmark) and commercial peanut extract (Stallergenes, Paris, France), with 1% histamine as a positive control.

#### BATs

BATs were performed, as described previously.^24^ In brief, heparinized whole blood (100 μL) from sensitized subjects was incubated with peanut allergen extract (ALK-Abelló) or anti-IgE (0.5 μg/mL) in a 37°C water bath for 15 minutes. Cells were immunostained with anti-human CD3, CD303, CD294 (chemoattractant receptor–homologous molecule expressed of T_H_2 cells [CRTH2]), CD203c, CD63, and CD107a (all from BD Biosciences). Erythrocytes from whole blood were lysed with BD lysing solution (BD Biosciences) for 10 minutes at room temperature in the dark, samples were centrifuged (for 5 minutes at 200*g*), and supernatants were discarded. The resulting cell pellets were washed in 3 mL of PBS (without Ca^2+^ and Mg^2+^) and resuspended in 450 μL of ice-cold fixative solution (CellFix; BD Biosciences) before acquisition on the BD FACSCanto II flow cytometer. Nonactivated and activated basophils were identified as CD203c^dim^CRTH2^+^ and CD203c^bright^CD3^−^CD303^−^CRTH2^+^ cells, respectively. Additionally, activated cells were also identified as CD63^+^ and CD107a^+^CD3^−^CD303^−^CRTH2^+^ basophils. Analyses were performed with BD FACSDiva software (version 6.1.1; BD Biosciences).

### Data and statistical analysis

#### Threshold sensitivity calculation

A 4-parameter logistic regression model (with Hill slope) was used to fit the dose-response curve and estimate the half-maximal effective concentration (EC_50_) for each patient. Threshold sensitivity (CD-sens), the inverse of the half-maximal effective allergen concentration multiplied by 100 (CD-sens = [1/EC_50_] × 100) was then calculated, as described previously by Johansson et al.^25^ Higher CD-sens values indicate greater sensitivity.

To best represent the MAT response as a single number, we calculated the area under the curve (AUC) using the trapezoidal rule on logarithmically transformed venom concentrations, as previously described.^26^ Statistical analyses (except receiver operating characteristic [ROC] curve analyses) were performed with R software and its affiliated software packages. Data are represented as medians and interquartile ranges and were compared by using a Mann-Whitney *U* test. A 2-sided *P* value of less than .05 was considered statistically significant. Correlation coefficients were calculated by using the Spearman R test in Prism software (version 7; GraphPad Software, La Jolla, Calif). Intraclass correlation (ICC) was calculated in R software to assess MAT and BAT reproducibility. We used ICC rather than coefficient of variation because the former is a more appropriate measure of interassay variation where there is no natural zero point.^27^ ROC curves and associated parameters were determined with Prism software.

#### Functional data analysis

To identify distinct response profiles and their characteristics, we performed an exploratory analysis on the trajectories defined by the MAT measurements (details are provided in the Methods section in this article's Online Repository). To uncover the dynamic of the latent allergic response process, we examined the discrete trajectories in a continuous way using functional data analysis (FDA).^28^ All of the FDAs were carried out in the MATLAB language using the toolbox for FDA. We then undertook FDA of the MATs. To mitigate the effect of the unequal intervals between allergen concentrations, we applied a logarithmic transformation of the form log(x+c), with c=0.001. For each patient, 6 measurements obtained through the MAT assay were converted into continuous curves by using B-spline basis functions.^28^ The resultant fitted curves formed the basis for subsequent analyses.

To identify the dominant modes of variation of the response patterns, we applied functional principal component (FPC) analysis to the fitted curves.^28^ We then used k-means clustering to estimate distinct response patterns. To determine the optimal number of clusters, we used several evaluation measures available through the R package NbClust.^29^ Further details of analyses can be found in the Methods section in this article's Online Repository.

## Results

### MAT development

#### Generation of hMCs from peripheral blood precursors

**Fig E1:**
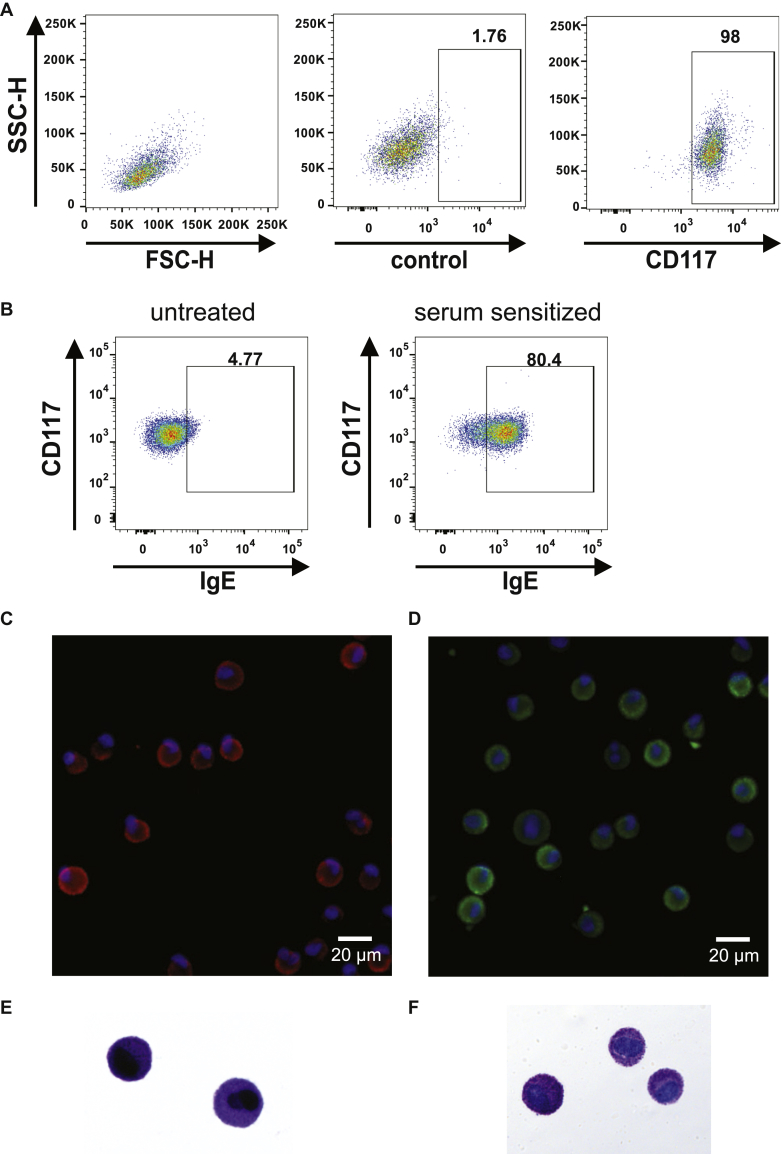
Characterization of hMCs. **A,** After 8 to 10 weeks of culture, hMC maturation was controlled by measuring the size granularity, (side scatter *[SSC]*–H/forward scatter *[FSC]*–H) and CD117 surface expression by using flow cytometry. Control shows fluorescence minus one values for CD117 control staining. **B,** Serum IgE binding. hMCs were sensitized overnight with human serum (1:10 dilution) or not for control hMCs and washed. IgE fixed on the surfaces of hMCs was detected by using flow cytometry. Numbers represent percentage cells in the gate. **C** and **D,** Immunofluorescence for tryptase (Fig E1, *C*) and chymase (Fig E1, *D*). A spectrum of tryptase and chymase expression is seen within the MC population. **E** and **F,** MC metachromatic granules were identified by using May-Grünwald Giemsa (Fig E1, *E*) or toluidine blue (Fig E1, *F*) staining.

After 8 to 10 weeks of culture, hMCs derived from peripheral blood precursors had the phenotypic and functional properties of mature hMCs: they expressed CD117^+^ (see Fig E1, *A*, in this article's Online Repository at www.jacionline.org) and surface IgE receptors that bound strongly to serum IgE (see Fig E1, *B*). We confirmed the presence of tryptase and chymase using immunofluorescence (see Fig E1, *C* and *D*), with characteristic granularity patterns after staining with Giemsa and toluidine blue (see Fig E1, *E* and *F*).

#### Sensitized hMCs are highly sensitive to allergen-induced degranulation

**Fig 1:**
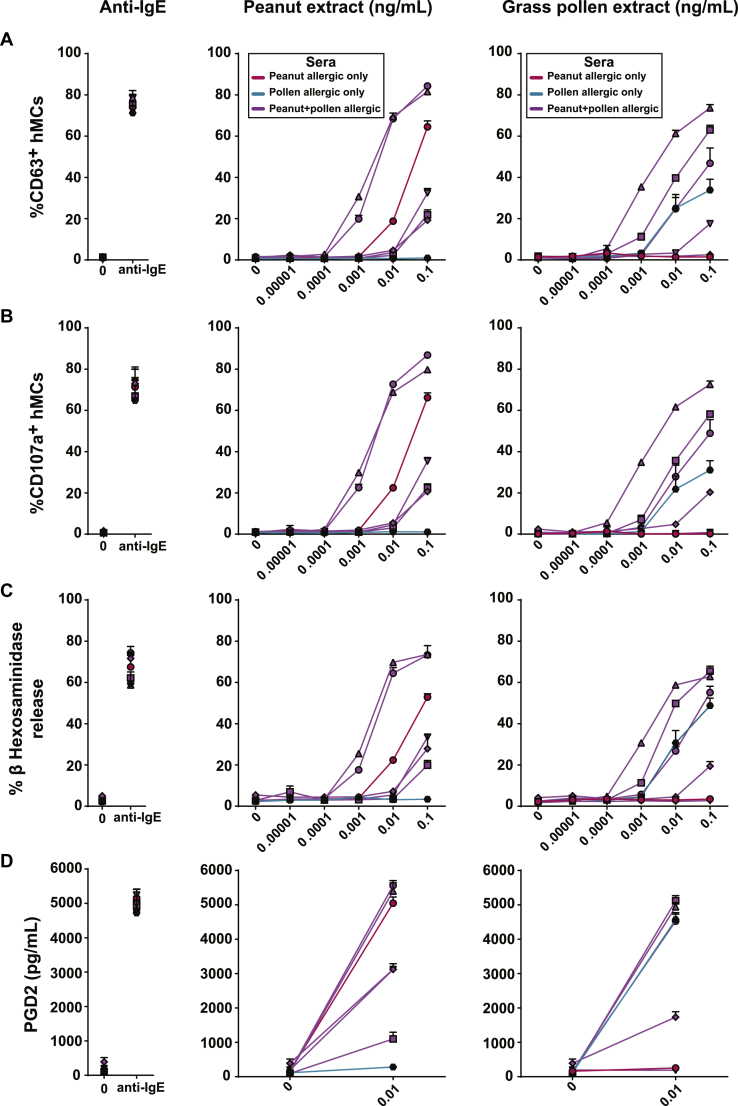
Peanut- and grass pollen–induced degranulation of hMCs sensitized with sera of patients with peanut and grass pollen allergy (sensitized to both peanut and pollen, n = 5; sensitized to peanut only, n = 1; sensitized to grass pollen but not peanut, n = 1). hMCs were sensitized overnight with sera from patients with peanut allergy, patients with grass pollen allergy, or both; washed; and either left untreated or exposed for 1 hour to anti-IgE (10 μg/mL) as a positive control (*left*), different concentrations of peanut extract *(middle)*, or grass pollen extract *(right)*. **A** and **B,** hMC degranulation was measured based on CD63 (Fig 1, *A*) and CD107a (Fig 1, *B*) surface staining and analyzed by using flow cytometry. **C,** β-Hexosaminidase levels were measured in cell pellets, as well as in supernatants. Percentage β-hexosaminidase release is shown. **D,** PGD_2_ levels were measured in supernatants. The assay was performed in duplicates or triplicates with pooled hMCs from at least 3 different healthy donors. *Symbols* indicate individual patients, and values indicate means ± SDs.

**Fig E2:**
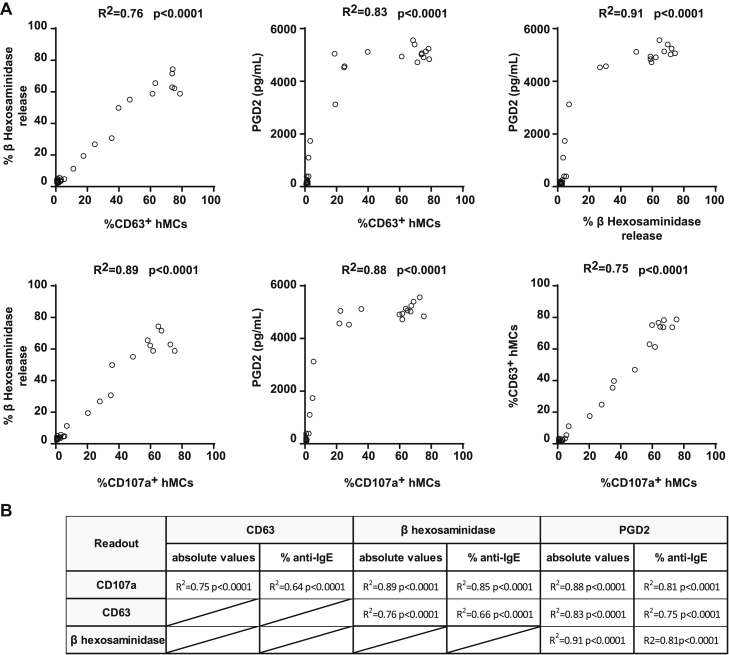
Correlation of surface expression and mediators release measurement. hMCs were sensitized overnight with serum from patients with peanut allergy, patients with grass pollen allergy, or both (volume ratio, 1:10); washed; and either left untreated or exposed for 1 hour to different concentrations of peanut or grass pollen extract or anti-IgE (10 μg/mL) as a positive control. hMC degranulation was measured by CD63 and CD107a surface staining and analyzed by using flow cytometry. β-Hexosaminidase levels were measured in cell pellets, as well as in supernatants, and expressed as percentage β-hexosaminidase release. PGD_2_ levels were measured in supernatants. The assay was performed in duplicates or triplicates with pooled hMCs from at least 3 different healthy donors. Correlations were calculated by using Spearman *R (R*^*2*^*)* values (**A**) with both absolute values and percentages of readout induced by anti-IgE summarized in **B**.

We passively sensitized primary hMCs using sera from patients with peanut and pollen allergy. To assess their degranulation after stimulation with peanut and grass allergen extract, we measured surface expression of CD63 and CD107a (lysosomal-associated membrane protein 1) using flow cytometry^30^ and release of β-hexosaminidase (from intracellular granules) and PGD_2_ (secreted *de novo* after MC activation). *In vitro* incubation with allergen resulted in a dose-dependent increase in CD63 and CD107a membrane expression (Fig 1, *A* and *B*) and β-hexosaminidase release (Fig 1, *C*); all immunologic readouts correlated significantly (see Fig E2 in this article's Online Repository at www.jacionline.org). Functional degranulation was further confirmed by the observation that allergen stimulation caused allergen-specific release of PGD_2_ (Fig 1, *D*). Incubation with 10 μg/mL anti-IgE (as a positive control) resulted in a similar degree of degranulation (Fig 1). The hMC response was allergen specific, and there was no evidence of hMC activation or degranulation when we used sera from patients sensitized to allergens other than that used for stimulation.

**Fig E3:**
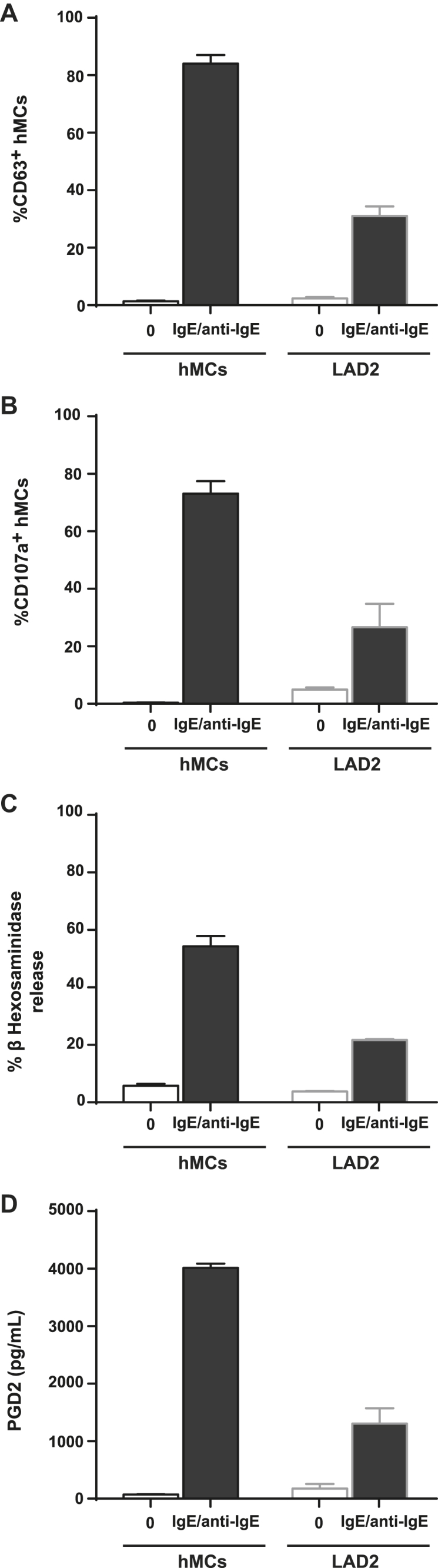
hMCs are more susceptible to IgE-mediated degranulation than LAD2 cells. hMCs were sensitized overnight with 1 μg/mL human IgE washed and either left untreated (0 μg/mL) or exposed for 1 hour to anti-IgE (10 μg/mL). **A** and **B,** hMC degranulation was measured based on CD63 (Fig E3, *A*) and CD107a (Fig E3, *B*) surface staining and analyzed by using flow cytometry. **C,** β-Hexosaminidase levels were measured in cell pellets, as well as in supernatants. Percentage of β-hexosaminidase release is shown. **D,** PGD_2_ levels were measured in supernatants. The assay was performed with pooled hMCs from at least 3 different healthy donors.

Stimulation with anti-IgE resulted in greater surface expression of CD63 and CD107a and higher levels of β-hexosaminidase and PGD_2_ release in hMCs compared with LAD2 cells (see Fig E3 in this article's Online Repository at www.jacionline.org).

In summary, hMCs passively sensitized with sera from donors with peanut and/or pollen allergy were very sensitive to low doses of allergen. The sensitized hMCs demonstrated allergen-specific and dose-dependent degranulation by using both expression of surface activation markers and functional assays, indicating that hMCs are suitable as primary effector cells for screening studies. Given the correlation between immunologic parameters, we used CD63 expression as the readout in subsequent experiments.

#### Application of MATs in patients with peanut and wasp venom allergy

##### Peanut allergy

**Fig 2:**
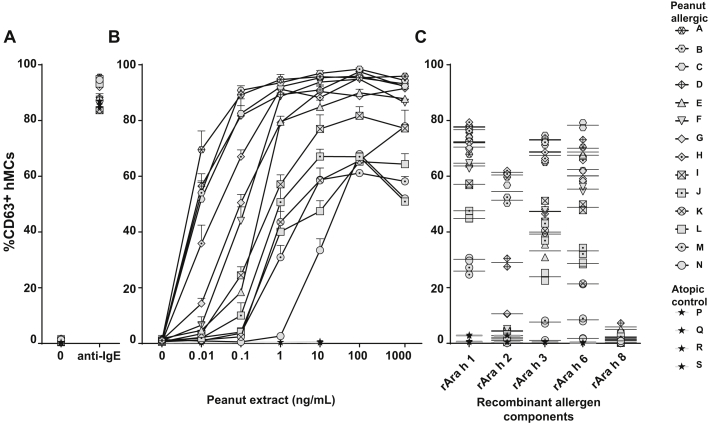
hMC degranulation using hMCs from patients with peanut allergy. hMCs were sensitized overnight with sera of patients with peanut allergy or control sera (volume ratio, 1:10), washed, and either left untreated or exposed for 1 hour to anti-IgE (10 μg/mL; **A**), different concentrations of peanut extract (as indicated; **B**), or recombinant peanut allergens (rAra h 1, rAra h 2, rAra h 3, rAra h 6, and rAra h 8 [10 nmol/L]; **C**). hMC degranulation was measured by using CD63 surface staining and analyzed with flow cytometry. Fig 2, *A* and *B*, show one representative experiment of 2, and the assay was performed in duplicates or triplicates with pooled hMCs from at least 3 different healthy donors. Values indicate means ± SDs, and *symbols* show individual subjects. In Fig 2, *C*, the assay was performed in duplicates; *lines* indicate the mean of replicates, and *symbols* show each subject.

**Fig 3:**
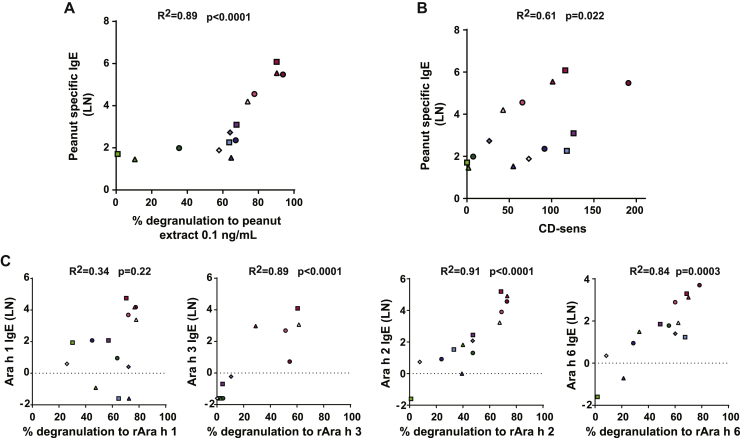
Correlation of serum *Arachis hypogaea* IgE measurements and hMC degranulation analysis in the panel of 14 patients with peanut allergy. Correlation of serum specific peanut IgE levels versus peanut extract–induced hMC degranulation **(A)**, serum specific peanut IgE levels versus MC allergen CD-sens **(B)**, and serum IgE levels specific for rAra h 1, rAra h 3, rAra h 2, and rAra h 6 versus hMC degranulation induced by the respective recombinant peanut allergen **(C)** are shown. IgE levels are expressed as natural logarithm *(LN)*. Measurements noted as less than the limit of detection (<0.4) in Table E1 were given a value of half the limit of detection (0.2). CD-sens values were calculated as described previously by Johansson et al,^25^ with higher values implying greater sensitivity. *Symbols* indicate mean values for each investigated serum from patients with peanut allergy. Degranulation was measured based on surface expression of CD63 on hMCs by flow cytometry. Ara h IgE levels are expressed in natural logarithm *(LN)*. Measurements noted as less than the limit of detection (<0.4) in Table E1 were given a value of half the limit of detection (0.2). *Symbols* indicate mean values for each investigated serum from patients with peanut allergy.

Primary hMCs were sensitized passively with sera from 14 patients with peanut allergy and 4 atopic control subjects without peanut allergy. All 14 patients with peanut allergy had a recent history of peanut-induced anaphylaxis (see Table E1 in this article's Online Repository at www.jacionline.org). Incubation of passively sensitized hMCs with increasing concentrations of peanut extract resulted in a dose-dependent expression of CD63 in patients with peanut allergy but not in atopic control subjects (Fig 2, *B*). Anti-IgE induced a similar degree of CD63 expression (Fig 2, *A*). There was a significant correlation (*R*^*2*^ = 0.89, *P* < .0001) between the level of hMC degranulation induced at 0.1 ng/mL peanut extract and the peanut-specific IgE titer (Fig 3). The CD-sens of the MAT^25^ showed a weaker correlation with peanut-specific IgE levels, with the patient population appearing to separate into 2 groups (Fig 3, *B*).

Stimulation of hMCs (sensitized with sera from patients with peanut allergy) with the recombinant peanut proteins rAra h 1, rAra h 2, rAra h 3, and rAra h 6 also increased CD63 expression (Fig 2, *C*). The Bet v 1 homologue rAra h 8 (implicated in pollen-food allergy syndrome rather than primary allergy to peanut) did not induce substantial hMC degranulation in these patients (Fig 2, *C*). Fig 3, *C*, shows the correlation between IgE titers to allergen components and hMC degranulation.

##### Wasp allergy

**Fig E4:**
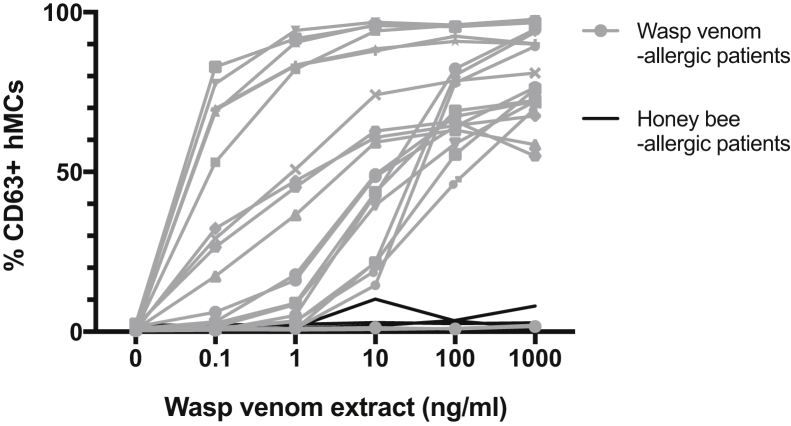
hMC degranulation in patients with a clinical history of systemic reaction to wasp (n = 21) or honeybee (n = 6) venom. hMCs were sensitized overnight with patients' sera, washed, and then incubated with varying concentrations of wasp venom extract (as indicated) for 1 hour. hMC degranulation was measured based on CD63 surface expression by using flow cytometry.

hMCs were sensitized by using sera from 21 patients with a confirmed systemic reaction to wasp venom and 7 patients with previous systemic reaction to honeybee but not wasp venom (see Table E2 in this article's Online Repository at www.jacionline.org). Incubation of passively sensitized hMCs with increasing concentrations of wasp venom extract resulted in a dose-dependent expression of CD63 in patients with wasp venom allergy but not those with honeybee venom allergy (see Fig E4 in this article's Online Repository at www.jacionline.org).

### Validation, diagnostic performance, and comparison of MATs with other diagnostic tests

#### Diagnostic cutoff values for MATs in patients with peanut allergy

We performed MATs in a further cohort of 42 peanut-sensitized patients before they underwent DBPCFCs to peanut. Demographic and clinical characteristics of the study population are shown in Table I; 30 participants reacted to DBPCFCs and were classified as having peanut allergy, whereas 12 passed the challenge without experiencing dose-limiting symptoms and were categorized as sensitized but peanut tolerant.

**Table I:** Demographic and clinical characteristics of patients assessed for peanut allergy

	Patients with peanut allergy (n = 30)	Peanut-sensitized but tolerant subjects (n = 12)	*P* value
Age (y)	13.5 (11-17)	17.5 (9-29)	.36
Male sex (%)	50	75	
SPT response to peanut (mm)	10 (7-12)	7 (5-9)	**.02**
IgE to peanut (kU_A_/L)	26 (5.5-85)	0.35 (0.3-2.0)	**<.001**
IgE to Ara h 1 (kU_A_/L)	3.9 (<0.1-27)	<0.1 (<0.1-0.23)	**<.001**
IgE to Ara h 2 (kU_A_/L)	13.6 (3.1-80)	0.2 (<0.1-0.6)	**<.001**
IgE to Ara h 3 (kU_A_/L)	0.45 (<0.1-7.0)	<0.1 (<0.1-0.1)	**<.001**
IgE to Ara h 8 (kU_A_/L)	<0.1 (<0.1-6.0)	0.29 (<0.1-2.9)	.35
Reaction severity at DBPCFC	Mueller grade 1: 6 (20%)		
	Mueller Grade 2: 9 (30%)	NA	
	Mueller Grade 3: 15 (50%)		

Data are expressed as medians (interquartile ranges). *P* value refers to a comparison between patients with peanut allergy and peanut-tolerant subjects using the Mann-Whitney test. Boldface indicates *P* < .05.

*NA*, Not available.

**Fig E5:**
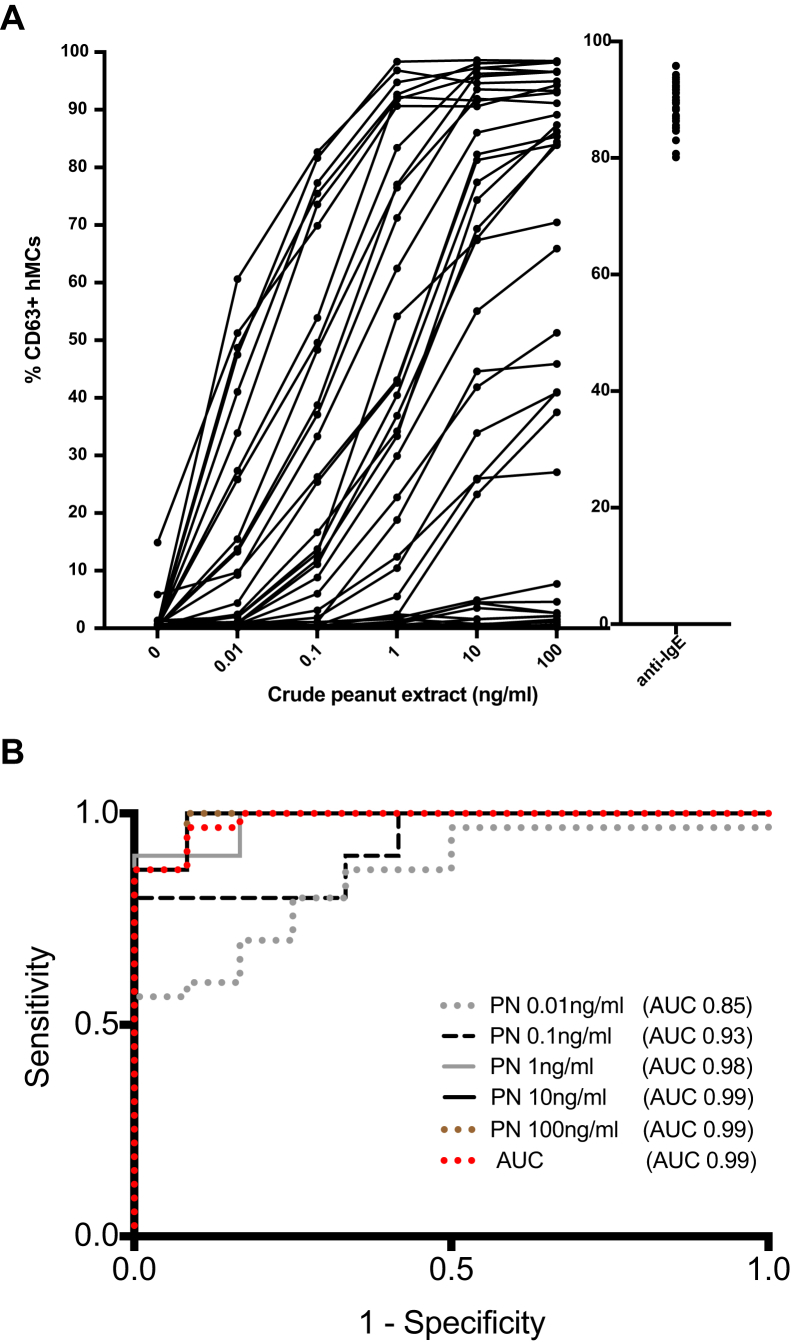
hMC degranulation in peanut-sensitized patients from the validation cohort (n = 42). **A,** hMCs were sensitized overnight with patients' sera, washed, and then incubated with varying concentrations of peanut extract (as indicated) or anti-IgE (10 μg/mL) for 1 hour. hMC degranulation was measured based on CD63 surface expression by using flow cytometry. **B,** Corresponding ROC curve. *AUC*, Area under curve; *PN*, peanut.

Individual MAT dose-response curves are shown in Fig E5, *A*, in this article's Online Repository at www.jacionline.org. By using ROC curve analysis, the MAT outcome measures with the best performance to discriminate patients with peanut allergy from peanut-tolerant patients were MAT response to crude peanut at 10 and 100 ng/mL concentrations and AUC for the dose-response curve (MAT-AUC; all AUC, 0.99; see Fig E5, *B*). Therefore we used MAT-AUC as the outcome measure for further analyses.

#### Test performance characteristics of MATs compared with existing diagnostics for peanut allergy

**Fig 4:**
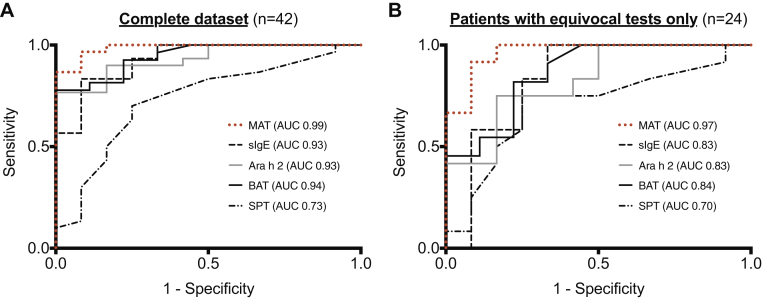
ROC curves comparing discrimination performance of the MAT with that of other diagnostic modalities in 42 peanut-sensitized subjects, of whom 30 reacted to less than 4.4 g of peanut protein with objective symptoms **(A)**, and a subgroup of peanut-sensitized subjects with equivocal results for conventional allergy tests **(B)**. In both scenarios the MAT had the most favorable diagnostic accuracy compared with other tests in identifying those with clinical reactivity to peanut.

All 42 patients in the validation cohort underwent conventional allergy testing (SPTs and sIgE measurement to peanut), as well component testing and BATs. We assessed the performance characteristics (sensitivity and specificity) for each test by using DBPCFCs as the reference. The study team was blinded to the results of the diagnostic tests at the time of challenge to prevent bias. By using ROC curve analysis, MATs had the most favorable discrimination performance (AUC, 0.99) compared with the other diagnostic tests (Fig 4, *A*, and Table II).

**Table II:** Discrimination performance of different diagnostic tests for peanut allergy in the principle study population (n = 42) and a subcohort of participants with equivocal tests (SPT or sIgE measurement, n = 24)

Population	Diagnostic tests	Optimal cutoff∗	AUC ROC (95% CI)	Sensitivity (95% CI)	Specificity (95% CI)	PLR (95% CI)	NLR (95% CI)	Odds ratio (95% CI)
Whole population (n = 42)	SPT (mm)	8	0.73 (0.56-0.90)	70 (50-85)	75 (43-95)	2.8 (1.0-7.7)	0.08 (0.08-0.41)	7 (1.5-32.1)
	sIgE to peanut (kU_A_/L)	3.8	0.93 (0.85-1.0)	83 (65-94)	92 (62-100)	10 (1.5-66)	0.18 (0.08-0.41)	55 (5.7-527)
	sIgE to Ara h 2 (KU_A_/L)	1.64	0.93 (0.86-1.0)	77 (58-90)	83 (52-98)	4.6 (1.3-16)	0.28 (0.14-0.56)	16.4 (2.9-93)
	BAT, % CD63	7.8	0.94 (0.87-1.0)	80 (61-92)	89 (52-100)	7.2 (1.1-46)	0.22 (0.11-0.48)	32 (3.3-308)
	MAT-AUC	6.3	0.99 (0.96-1.0)	97 (83-100)	92 (62-100)	11.6 (1.8-76)	0.04 (0.01-0.25)	319 (18.3-5556)
Subgroup with equivocal SPT/sIgE results (n = 24)	SPT (mm)	8	0.70 (0.48-0.90)	75 (50-85)	75 (43-95)	3.0 (1.1-8.4)	0.33 (0.12-0.94)	9 (1.4-57)
	Specific IgE (KU_A_/L)	0.43	0.83 (0.65-1.0)	100 (74-100)	67 (62-100)	3.0 (1.4-6.7)	0	∞
	Ara h 2 (KU_A_/L)	0.72	0.83 (0.66-0.99)	75 (43-95)	83 (52-98)	4.5 (1.2-16)	0.30 (0.11-0.83)	15 (2.0-111)
	BAT, % CD63	10.5	0.84 (0.67-1.0)	83 (52-98)	78 (40-97)	3.8 (1.1-13)	0.21 (0.06-0.80)	17.5 (2.0-156)
	MAT-AUC	6.3	0.97 (0.90-1.0)	92 (62-100)	92 (62-100)	11.0 (1.7-72)	0.09 (0.01-0.60)	121 (6.7-2188)

*NLR*, Negative likelihood ratio; *PLR*, positive likelihood ratio.

∗ Optimal cutoffs were determined by using the Youden index.

We undertook a further analysis in a subgroup of 24 peanut-sensitized patients with equivocal conventional testing (peanut SPT response < 8 mm or sIgE levels < 15 kU_A_/L),^31^ 12 of whom had a positive DBPCFC result (see Table E3 in this article's Online Repository at www.jacionline.org). In this subgroup of patients, MATs continued to trend toward superior discrimination performance compared with other diagnostic tests (Fig 4, *B*, and Table II).

#### Reproducibility of MATs compared with BATs

MAT responses in the validation cohort were assessed on at least 2 separate occasions in 25 patients with peanut allergy who also underwent BATs. We calculated ICC (ie, reproducibility of MATs on the same sample on different occasions by using different pooled hMCs). We used ICC rather than coefficient of variation because the former is a more appropriate measure of interassay variation where there is no natural zero point.^27^ Overall, the ICC for MATs was 0.96 (95% CI, 0.95-0.97). In contrast, the ICC for BATs performed on the same patients on 2 separate occasions up to 4 weeks apart was 0.43 (95% CI, 0.27-0.57).

#### Exploratory analysis to identify patterns of response in MATs

**Fig 5:**
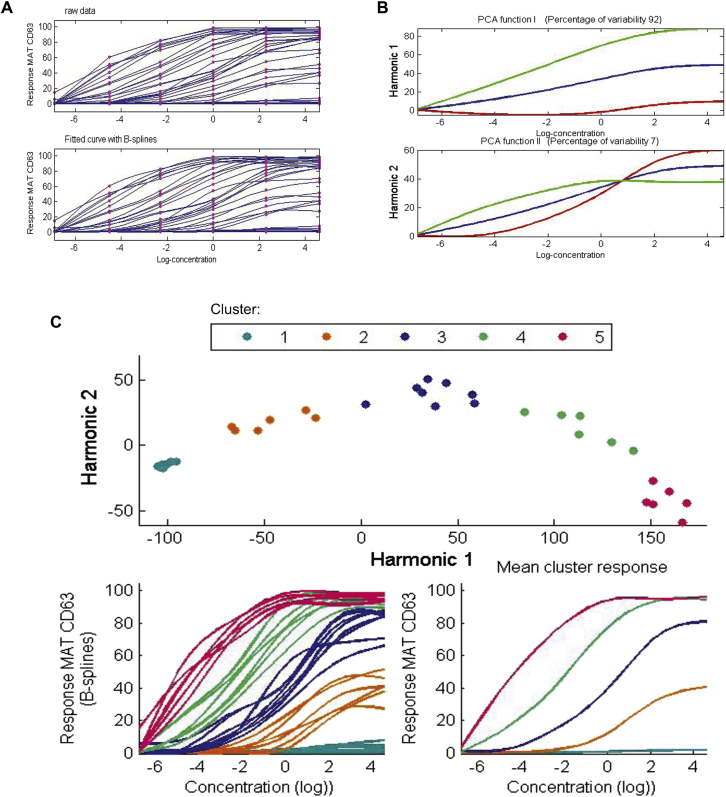
FDA of the MAT. **A,** Raw data and smoothed curves for peanut-sensitized subjects (n = 42). **B,** Principal components analysis *(PCA)* of pattern response measured by using the MAT assay. *Lines* show the effect of adding *(green line)* or subtracting *(red line)* 2 SDs from the mean response curve *(blue line)*. **C,** Distinct clusters of allergic responses to peanut obtained through k-means on FPC scores.

**Fig E6:**
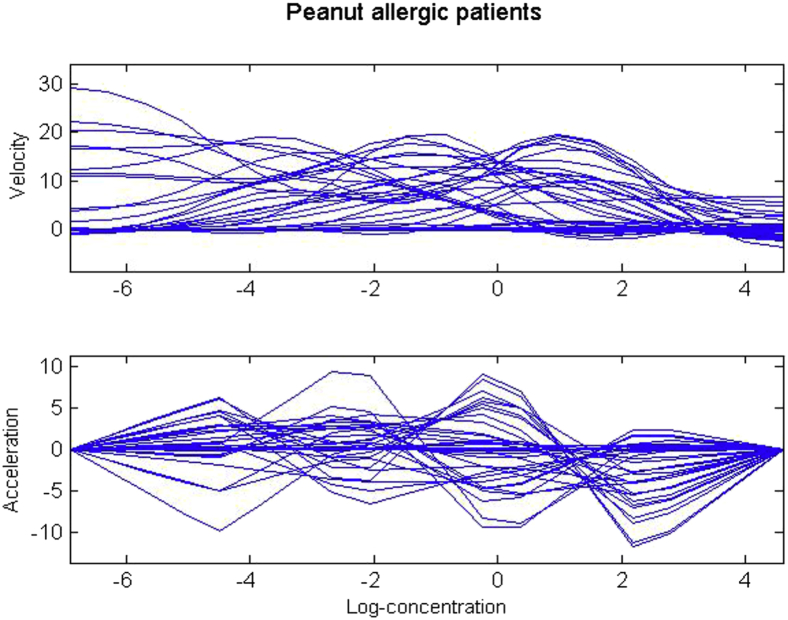
First and second derivatives of response curves for peanut-sensitized patients.

We used data-driven analyses to identify whether there were subgroups of patients with similar MAT responses among the total cohort of 42 peanut-sensitized patients.^28^ Fig 5, *A*, shows the MAT dose response for each patient with individually fitted smooth response curves. A large variation between individual curves was observed, particularly at high allergen concentrations. The FDA^28^ indicated the presence of distinct groups characterized by different velocity and acceleration (see Fig E6 in this article's Online Repository at www.jacionline.org).

**Fig E7:**
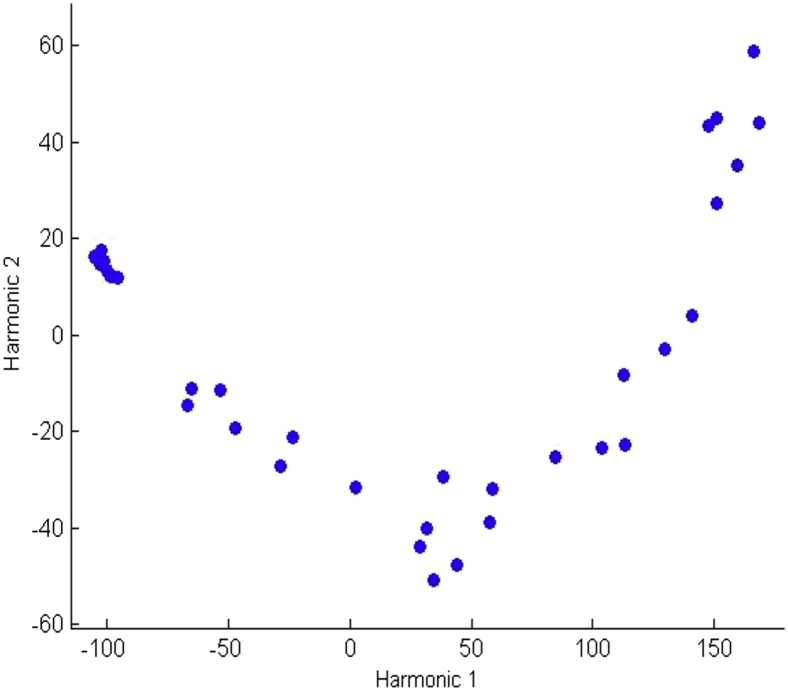
Scores of patients on the first 2 principal components of the allergic response.

We identified the dominant modes of variation of response patterns using FPC analysis.^28^ The first FPC explained 92% of the variation, and the second FPC explained 7% (Fig 5, *B*, and see Fig E7 in this article's Online Repository at www.jacionline.org). The first FPC represented the overall level of allergic response, with larger effects registered for increasing allergen concentration and with low, moderate, and high responses evident. The second FPC reflected response changes, which we interpreted as the sensitivity to the specific allergen concentration. In general, patients had a steadily increasing response across allergen concentrations. Variations revealed a group with high sensitivity to lower doses and a group requiring higher doses to induce a response.

**Fig E8:**
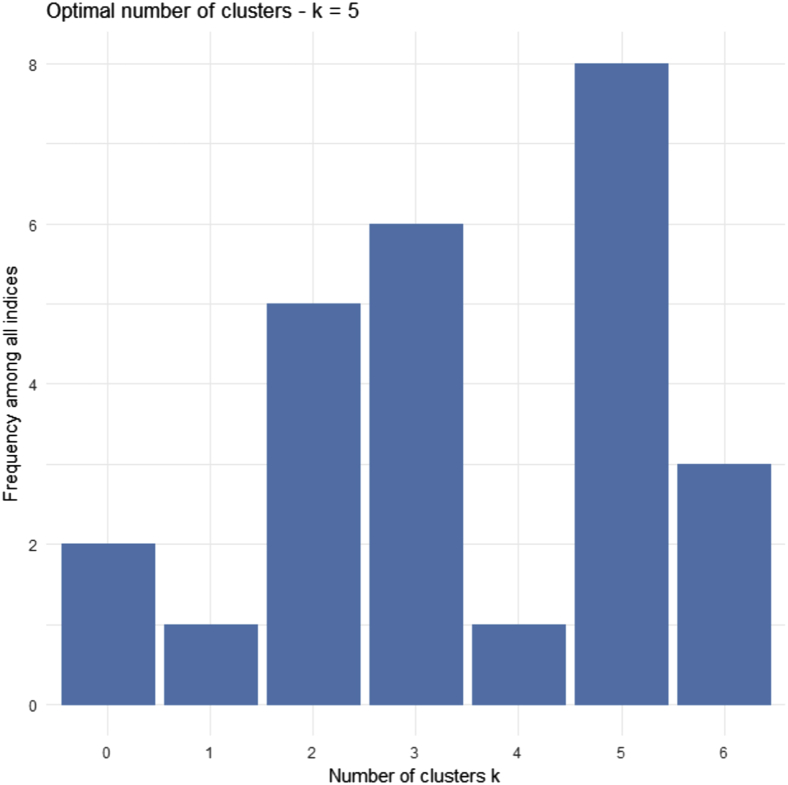
Evaluation measures distribution for the choice of the optimal number of cluster obtained with the package NbClust.

**Fig E9:**
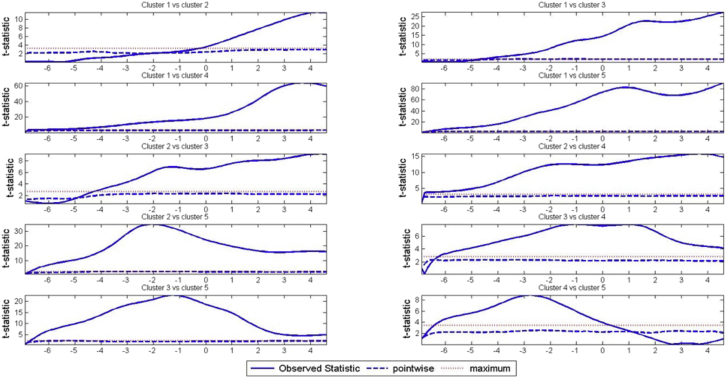
Functional *t* test for differences between responses in the 5 clusters.

**Fig E10:**
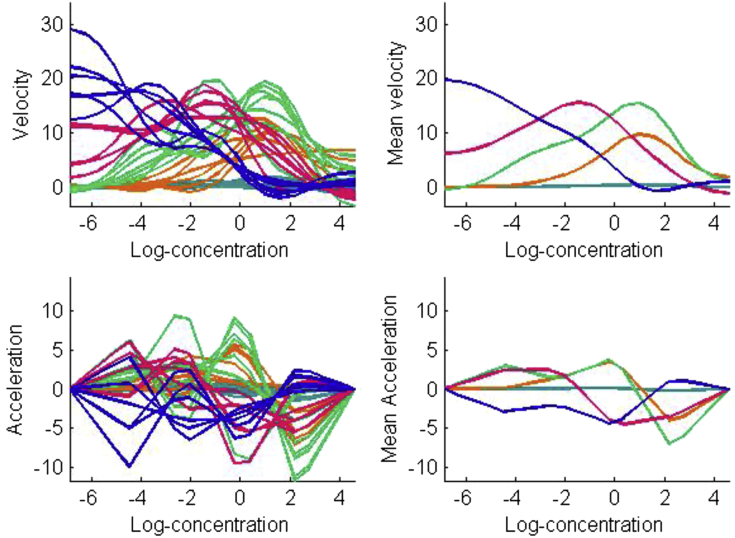
Velocity and acceleration in the distinct response patterns in the clusters.

To further investigate the structure of the data, we performed k-means clustering.^32^ Results indicated a well-defined partition of the response patterns, with 5-cluster solutions being optimal (see Fig E8 in this article's Online Repository at www.jacionline.org). The clusters differed significantly from one another (functional *t* tests, see Fig E9 in this article's Online Repository at www.jacionline.org). Cluster 1 (n = 16) was characterized by no response or low response, and velocity and acceleration were stable throughout the entire range of concentrations; this group included peanut-tolerant patients. In contrast, patients in cluster 5 (n = 6) had a high response to low doses and quickly reached a response peak, which then became constant for the remaining concentrations (Fig 5, *C*, and see Fig E10 in this article's Online Repository at www.jacionline.org). Clusters 2 to 4 were characterized by distinct levels of sensitivity (details are provided in the Methods section in this article's Online Repository at www.jacionline.org). When we related the clusters to clinically defined severity of peanut allergy ascertained by DBPCFCs, cluster 1 corresponded to sensitized patients who were either nonreactive or experienced symptoms only at relatively high levels of exposure (>4.4 g peanut protein, approximately 20 peanuts), although the higher clusters corresponded to patients who reacted to far lower levels of exposure with a tendency toward more significant systemic reactions (see Table E4 in this article's Online Repository at www.jacionline.org).

**Fig 6:**
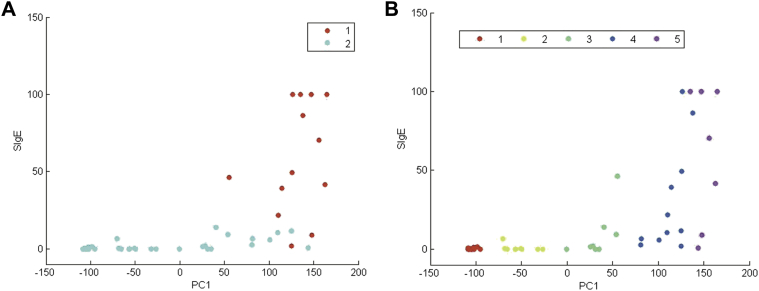
K-means cluster on sIgE data in patients with peanut allergy. **A,** IgE clusters on peanut-specific IgE versus the first FPC. **B,** MAT clusters on peanut-specific IgE versus the first FPC.

**Fig E11:**
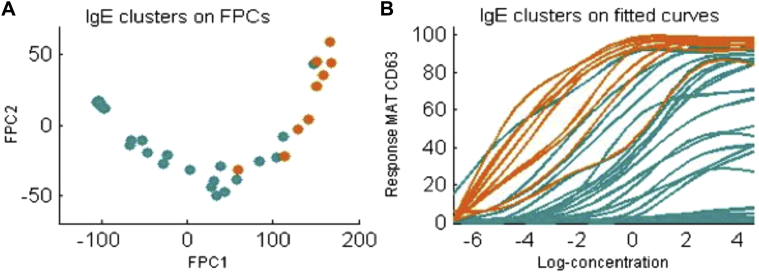
K-means clustering on sIgE data in patients with peanut allergy. **A**, MAT responses on the first 2 principle components, assigned to the 2 IgE clusters detected. **B**, IgE clusters on MAT response curves to peanut.

To compare the discriminatory power of sIgE measurement in comparison with the MAT, we also undertook k-means clustering on sIgE data (see Fig E11 in this article's Online Repository at www.jacionline.org). The optimal number of clusters was 2, with the clustering distinguishing between patients with low versus those with high sIgE levels. When we compared the 2 partitions by plotting patients on the space defined by variable sIgE levels and the first FPC (relating to MAT the response), the response for MAT clusters 4 and 5 appeared to be independent of sIgE levels (Fig 6).

## Discussion

We developed a robust and reproducible MC-based assay to improve the diagnosis of IgE-mediated allergy using human MCs derived from human progenitor cells. hMCs sensitized with sera from patients with peanut, grass pollen, and Hymenoptera (wasp venom) allergy demonstrated allergen-specific and dose-dependent degranulation by using both expression of surface activation markers (CD63 and CD107a) and functional assays (PGD_2_ and β-hexosaminidase release). The MAT is a very sensitive assay, with significant levels of surface expression of CD63 activation markers after stimulation with peanut at concentrations up to 2-log lower than that required for the BAT.^15^ We have shown that in the cohort of peanut-sensitized patients who underwent DBPCFCs to peanut, our novel MAT appeared to confer superior diagnostic accuracy compared with existing diagnostics in distinguishing between patients with clinical reactivity and those who did not react to DBPCFCs.

Our data imply that the MAT response is not just dependent on serum specific IgE levels. When we compared the partitions obtained by using k-means clustering on sIgE data with that relating to MAT response, the latter appeared to be independent of sIgE (Fig 6, *D*). This is consistent with our observation of 2 separate groups of patients when comparing the MAT readout (measured by CD-sens, a marker of MC sensitivity): one group had a higher MAT sensitivity to the same level of sIgE (Fig 3, *B*). Thus the MAT response does not appear to depend exclusively on the concentration of sIgE levels, suggesting that hMC degranulation can be regulated by additional elements, such as affinity or a combination of allergen IgE specificities that vary between subjects.

In addition to diagnosis, other potential applications of the MAT could include investigations of the intracellular communication pathways and molecular mechanisms engaged in the IgE-mediated activation of these cells to allergen, the assessment of MAT responses to different allergen epitopes, and, given the very high sensitivity of MAT, assessment for the unintended presence of food allergens during food production, The MAT might therefore be useful as an aid to associated risk allergen management.

We found hMC cultures to be stable, reproducible, and highly sensitive (responding to concentrations of peanut <1 pg/mL); these characteristics mean they are ideal tools to investigate the unique effector functions of human MCs. Our protocol used culture media free of serum, thus reducing the risk of nonspecific patient-protein interactions. Some groups have attempted to standardize diagnostic methods by using cell lines (eg, humanized rat basophilic leukemic cells [RBL-2H3 cells] or human leukemic MCs [LAD2 cells]) that express FcεRI, the high-affinity IgE receptor.^33^, ^34^, ^35^

We found that stimulation of primary hMCs with anti-IgE resulted in more degranulation than that seen with LAD2 cells under the same conditions, suggesting that hMCs might be more suitable than LAD2 cells in FcεRI-mediated degranulation studies. Indeed, LAD2 cells, being of tumor origin, are slow growing in culture^36^ and unstable in that they eventually lose their capacity to undergo FcεRI-mediated degranulation,^37^ a key characteristic of MCs. Therefore LAD2 cells might not be representative of a typical hMC phenotype.

We also believe that hMCs are superior to rodent RBL-2H3 cells stably transfected with human FcεRI for diagnostic purposes. The latter is a humanized cell line, which in itself may be a shortcoming, and also shows variability in their IgE-binding capacity.^38^ A direct comparison between RBL-2H3 cells and primary human basophils showed no response from RBL-2H3 cells after sensitization with sera from a patient with chronic urticaria, despite primary basophils showing a strong response under the same conditions, further underlining the drawbacks of using this cell line for allergy testing.^39^

The stability, reproducibility, and higher sensitivity of hMCs recommend them as ideal tools to investigate the unique effector functions of human MCs and the intracellular molecular mechanisms and signaling pathways that distinguish human MCs from basophils in allergen reactivity.

A number of groups have sought to assess and validate the BAT for the diagnosis of peanut allergy.^15^, ^16^, ^17^ There are similarities in methodology between BATs and MATs, with both techniques using flow cytometry to assess the expression of surface activation markers after incubation with allergen *in vitro*. However, the BAT requires fresh blood, which is ideally processed within 4 hours of collection.^14^ Some groups have sought to perform BATs up to 24 hours after sampling, which results in downregulation of surface activation marker expression^40^; to date, the effect of this downregulation on diagnostic accuracy has not been assessed. The requirement to analyze fresh blood samples affects the feasibility of the BAT and has limited its use to a few specialist centers.^11^ Moreover, basophils from 6% to 17% of the population do not respond to IgE under standard BAT conditions, and BATs cannot be used for these subjects.^30^ In contrast, the MAT uses serum samples, which can be frozen and batch tested in a central facility. Although the differentiation of hMCs from blood progenitors requires time and specific expertise, this could take place in specialist centers, with the possibility of supplying hMCs to external laboratories. Our preliminary data, showing improved diagnostic performance of the MAT compared with other techniques, are convincing arguments to pursue further development of the MAT for clinical testing.

To ensure quality control across batches of hMCs, in each testing run we included an internal control and positive control (anti-IgE). Each batch was generated from 3 to 9 pooled donors, which reduces the risk of specific donor dependence and increases reproducibility. We confirmed this by assessing the MAT response on at least 2 separate occasions and assessing ICC, which was high. In contrast, the ICC for the BAT was much lower, an observation that likely represents the inherent biological variability in basophil reactivity from one day to another. One advantage of the BAT is that it evaluates both effector cell reactivity and serum factors (sIgE and IgG isotypes).^35^ However, in the context of food allergy, it is unclear as to the relative contributions to circulating basophils versus tissue-resident MCs.^18^ The observation that the MAT has better discrimination performance than the BAT implies that clinical reactivity/tolerance might depend more on serum factors (which are assessed by using the MAT) as opposed to basophil reactivity.

The peanut-sensitized patients used for the initial validation of the MAT are not representative of a general clinic population with indeterminate diagnostics, who might otherwise be selected to undergo a formal food challenge to clarify a diagnosis. We attempted to correct for this by including a subanalysis including only patients with indeterminate standard diagnostic tests (SPT response < 8 mm, sIgE level < 15 kU_A_/L).^15^ The results in this group of patients indicated that the MAT can confer improved diagnostic accuracy over existing allergy tests. However, further evaluation in a more representative clinic cohort is needed to confirm these findings.

Our exploratory data-driven analysis of MAT responses in patients with peanut allergy suggested that the patterns of response in the MAT can provide information relating to clinical reactivity, identifying the patients most at risk of significant anaphylaxis. If proved correct, this would be clinically very useful because no other diagnostic tests can predict the severity of the reaction on exposure.^41^ However, in this context our study generated a hypothesis that will require further studies to verify it.

In conclusion, we developed an MC-based assay to improve the diagnosis of IgE-mediated allergy that was robust and reproducible. Compared with other commonly used diagnostic tests, our novel MAT appeared to confer superior diagnostic accuracy in distinguishing between patients with true peanut allergy and those who are sensitized but tolerant to peanut.

**Key messages**

• We developed a robust and reproducible novel MC-based assay (by using primary human MCs generated from peripheral blood precursors).
• Compared with existing diagnostic tests, our novel MAT appeared to confer superior diagnostic accuracy in distinguishing between peanut-sensitized patients with and without clinical reactivity.

## Figures and Tables

**Table E1:** Serum IgE and IgG levels and associated clinical symptoms of the 14 patients with peanut allergy whose sera were used in the development of MATs

Patient no.	Total IgE (kU_A_/L)	Specific IgE (kU_A_/L) to:								sIgG to peanut (kU_A_/L)	Symptoms reported during accidental exposure
		Timothy grass	Peanut	Ara h 1	Ara h 2	Ara h 3	Ara h 6	Ara h 8	Ara h 9		
A	1300	ND	437	116	180	59.8	27.06	6.79	3.76	6.87	Vomiting, urticaria, ?loss of consciousness
B	460	20.6	95.1	40.2	49.5	14.7	18.03	0.4	<0.35	7.69	Lip angioedema, vomit × 2, throat tightness with vocal hoarseness, cough, dizziness
C	1400	ND	240	65.3	96.3	2.07	40.59	<0.35	<0.35	8.88	Generalized pruritus, mild angioedema, nausea, diarrhea, dyspnea with wheeze, dizziness
D	1500	>100	257	65.3	136	19.6	22.65	62.6	<0.35	10.3	Swollen mouth, tight throat, difficulty breathing, abdominal pain
E	1400	50.7	9.6	<0.35	4.6	<0.35	3.44	15.5	<0.35	2.75	Vomiting and abdominal cramps, chest tightness, and wheeze
F	55.4	<0.35	7.3	2.6	3.7	<0.35	5.96	<0.35	<0.35	<2	Facial angioedema and dyspnea
G	48.6	ND	15.3	1.5	8	0.8	4.06	<0.35	<0.35	3.72	Lip angioedema, throat tightness, nausea, and vomiting
H	190	19.5	66.7	29.6	25.2	21.4	6.78	<0.35	<0.35	8.18	Oral pruritus, dyspnea, pruritic rash over trunk
I	370	12.9	22.1	8	11.5	0.5	6.36	2.5	<0.35	4.3	Nausea, vomiting, dyspnea with chest tightness, loss of consciousness
J	430	1.5	4.3	0.4	6.2	<0.35	4.45	<0.35	<0.35	4.04	Nausea, chest pain, dysphagia and dyspnea, flushing
K	480	4.3	4.6	<0.35	1	<0.35	0.49	<0.35	21	4.27	Throat tightness, hoarse voice, dysphagia, nausea, and vomiting
L	81.6	11.3	6.6	1.81	2.09	<0.35	1.42	0.45	<0.35	6.06	Generalized urticaria with facial angioedema, abdominal cramps, chest tightness, and dizziness
M	140	ND	10.6	8	2.5	<0.35	2.58	<0.35	<0.35	<2	Dysphagia, dyspnea with wheeze, facial angioedema, generalized rash, abdominal cramps
N	1600	ND	5.5	6.97	<0.35	<0.35	<0.35	6.48	<0.35	3.07	Generalized urticaria, facial flushing, throat tightness, and wheezing

*ND*, Not determined.

**Table E2:** Patient demographics: Patients with venom allergy

	Wasp venom (n = 21)	Honeybee venom (n = 6)
Age (y)	41 (29-58)	46 (28-56)
Male (%)	66	100
IgE to wasp venom (kU_A_/L)	6.9 (3.1-22.8)	0.37 (0.35-0.89)
IgE to bee venom (kU_A_/L)	<0.35 (<0.35-1.8)	2.0 (1.4-6.1)
IgE to rVes v 5 (kU_A_/L)	21.7 (6.1-39.5)	<0.1 (<0.1-2.8)
IgE to rApi m 1 (kU_A_/L)	<0.1 (<0.1-0.35)	0.86 (0.40-2.5)
Reaction severity causing presentation	Mueller grade 1: 8 (28%)	Mueller grade 1: 0
	Mueller grade 2: 5 (17%)	Mueller grade 2: 2 (33%)
	Mueller grade 3: 5 (17%)	Mueller grade 3: 2 (33%)
	Mueller grade 4: 11 (38%)	Mueller grade 4: 2 (33%)

Data are expressed as medians (interquartile ranges).

**Table E3:** Patient demographics: Subpopulation with equivocal testing defined as either IgE sensitization to peanut equivalent to less than 95% positive predictive values for peanut allergy (IgE <15 kU_A_/L, SPT response < 8 mm)

	Patients with peanut allergy (n = 12)	Peanut-tolerant subjects (n = 12)	*P* value
Age (y)	13.5 (11-19)	17.5 (9-29)	.45
Male (%)	66	75	
SPT response to peanut (mm)	10 (7-12)	7 (5-9)	.10
IgE to peanut (kU_A_/L)	4.6 (1.7-7.4)	0.35 (0.3-2.0)	**.005**
IgE to Ara h 1 (kU_A_/L)	<0.1 (<0.1-0.22)	<0.1 (<0.1-0.23)	.49
IgE to Ara h 2 (kU_A_/L)	1.3 (0.4-3.9)	0.2 (<0.1-0.6)	**.006**
IgE to Ara h 3 (kU_A_/L)	<0.1 (<0.1-0.17)	<0.1 (<0.1-0.1)	.37
IgE to Ara h 8 (kU_A_/L)	<0.1 (<0.1-0.7)	0.29 (<0.1-2.9)	.62
Reaction severity at challenge	Mueller grade 1: 4 (33%)		
	Mueller grade 2: 3 (25%)	NA	
	Mueller grade 3: 5 (42%)		

Data are expressed as medians (interquartile ranges). *P* value refers to a comparison between patients with peanut allergy and peanut-tolerant subjects by using the Mann-Whitney test. Boldface indicates *P* < .05.

*NA*, Not available.

**Table E4:** Clinical characteristics of peanut-sensitized patients by clusters determined by using k-means clustering

	Cluster 1	Cluster 2	Cluster 3	Cluster 4	Cluster 5
No.	16	6	8	6	6
Age (y)	13.8 (11.4-22.8)	13.3 (12.0-14.8)	12.0 (9.0-17.7)	16.3 (13.4-24.8)	12.1 (11-13.8)
Male sex (%)	69	66	88	17	17
SPT response to peanut (mm)	7 (6-8)	10 (6-13)	8 (7-11)	13 (10-20)	11 (6-12)
IgE to peanut (kU_A_/L)	0.62 (0.32-2.4)	4.6 (2.6-7.5)	26.2 (12.8-34)	72.1 (42.6-95.7)	>100 (85 to >100)
IgE to Ara h 2 (kU_A_/L)	0.29 (0.21-0.87)	2.6 (0.9-3.8)	13.0 (8.8-19.3)	67.1 (26.5-95.3)	>100 (84 to >100)
Reaction severity at DBPCFC					
No reaction	75%	0%	0%	0%	0%
Mueller grade 1	6%	50%	0%	0%	33%
Mueller grade 2	0%	17%	63%	33%	17%
Mueller grade 3	19%	33%	37%	67%	50%
Eliciting dose at DBPCFC (peanut protein [g])	4.3 (4.3-8.4)	0.94 (0.44-1.9)	0.14 (0.043-0.29)	0.14 (0.066-0.14)	0.043 (0.043-0.12)

Data are expressed as medians (interquartile ranges).

## Methods

### Development of the MAT

#### Immunocytochemistry

hMCs were fixed in 4% paraformaldehyde in PBS for 10 minutes, allowed to settle on glass slides (Superfrost+; CellPath, Powys, United Kingdom), washed in PBS, and air-dried for a minimum of 1 hour. Cells were blocked in 10% normal goat serum (Vector Laboratories, Burlingame, Calif) diluted in PBS containing 0.1% Tween 20 (PBST) at room temperature for 30 minutes, followed by incubation with a mixture of anti-tryptase (ab2378; Abcam, Cambridge, United Kingdom; diluted 1:2000) and anti-chymase (ab186417; Abcam; diluted 1:2000) antibodies diluted in the blocking solution for 1 hour in a humid chamber at room temperature. Slides were washed in 3 changes of PBST over 9 minutes and incubated in a mixture of Alexa Fluor 488 anti-rabbit (ab150081; Abcam; diluted 1:200) and Alexa Fluor 555 anti-mouse (A-21424; Thermo Fisher Scientific; diluted 1:200) antibodies for 30 minutes in a humid chamber at room temperature. Slides were washed in PBST as above and mounted with glass coverslips by using Fluoroshield mountant containing 4′-6-diamidino-2-phenylindole dihydrochloride (ab104139; Abcam). Images were collected on an Olympus BX51 upright microscope (Olympus, Center Valley, Pa) by using a 20×/0.50 UPlanFln objective and captured using a CoolSNAP EZ camera (Photometrics, Tucson, Ariz) linked to MetaVue Software (Molecular Devices, Sunnyvale, Calif). Specific band pass filter sets for 4′-6-diamidino-2-phenylindole dihydrochloride, fluorescein isothiocyanate, and Texas red were used to prevent bleed through from one channel to the next. Images were then processed and analyzed with ImageJ software (http://rsb.info.nih.gov/ij; National Institutes of Health, Bethesda, Md).

### Allergen source

#### Peanut extract for MATs

Commercially available, lightly roasted, mechanically defatted peanut flour was obtained from Golden Peanut Company (Alpharetta, Ga). Samples were ground with a pestle and mortar and defatted by means of 2-stage addition of hexane (1:10 wt/vol) first for 3 hours and then overnight. Protein was extracted with 50 mmol/L Tris-HCl, pH 8.8 (1:50 wt/vol), for 1 hour with mixing at ambient temperature. Extracts were clarified by means of centrifugation (15,000*g* for 10 minutes), and protein was quantified by using the 2D-Quant assay kit (GE Healthcare, Buckinghamshire, United Kingdom). Confirmation of IgE binding to the extract was undertaken by using 1-dimensional PAGE and IgE immunoblotting.

#### Recombinant peanut allergens

The recombinant peanut allergens rAra h 1 (accession no. P43238), rAra h 3 (O82580), rAra h 2 (Q6PSU2), rAra h 6 (Q647G9), and rAra h 8 (B0YIU5) used in this study are recombinant proteins produced in *Escherichia coli* and were provided by EUROIMMUN AG. Timothy grass pollen allergen extract was kindly provided as well by EUROIMMUN AG. The peanut components had been tested according to quality process/criteria, including multiple steps, such as SDS-PAGE, Western blotting, mass spectroscopy, and a serologic test with reference sera.

#### Wasp venom

Wasp venom was sourced from Hal Allergy (Leiden, The Netherlands)

### Validation, diagnostic performance, and comparison of MATs with other diagnostic tests

#### SPTs

SPTs were performed to commercially available extracts (ALK-Abelló, Hørsholm, Denmark) by using single-point lancets, according to national guidelines. Histamine (10 mg/mL; Stallergenes) was used as a positive control. A positive SPT response was defined as a wheal size of at least 3 mm larger than that elicited by a saline control read at 15 minutes.

#### DBPCFCs to peanut

DBPCFCs were conducted according to international consensus criteria (PRACTALL).^E1^ In brief, subjects underwent double DBPCPCs over 2 separate days at least 14 days apart. On each day, subjects received increasing doses every 30 minutes of peanut protein (or placebo) at 3, 10, 30, 100, 300, 1000, and 3000 mg until stopping criteria were met (as per PRACTALL consensus). The order of DBPCFC challenges was determined by using a computer-generated randomization table (http://www.randomization.com). All members of the research team were blinded to the challenge assignment aside from the technician preparing the challenge material.

#### Measurement of IgE and IgG antibody levels

Levels of total IgE, peanut-specific IgE, and IgE to the allergen components rAra h 1, 3, 2, 6, 8, and 9 were measured by using ImmunoCAP (Thermo Fisher Scientific). IgG levels to peanut were measured with the Phadia 250 Laboratory System.

### FDA: Statistical learning and explanatory notes

#### Derivatives of response curves

In FDA the first and second derivatives provide useful insight into the dynamic of the investigated phenomenon. Fig E6 demonstrates the presence of distinct groups of responses with different velocity and acceleration. To further interpret these findings, we used the methodology of Silverman and Ramsay^E2^ to plot the overall mean function and the functions obtained by adding and subtracting a suitable multiple of the principal component function in question.

To better investigate the structure underlying the data, we plotted the principal component scores for pairs of harmonics to ascertain how the curves are distributed within the K-dimensional subspace spanned by the eigen functions (Fig E7).^E2^

#### Optimal number of clusters

The choice of the optimal number of clusters^E3^ has been based on 30 different evaluation indices. According to the majority rule, we have selected a 5-cluster solution (Fig E8).

#### Functional *t* test

Functional *t* tests^E2^ revealed that the clusters were significantly different from one another. There was little evidence of differences for clusters 1 and 2, 1 and 3, and 2 and 3 for low allergen concentrations. However, evidence of significant differences is found for higher doses (Fig E9). This is not surprising because the response curves in these clusters overlap for low doses, and the separation becomes evident after exposure to higher concentrations. On the contrary, clusters 4 and 5 differed only for low to moderate concentrations, whereas little evidence was found for higher doses.

#### Derivatives of the response curves in clusters

Applying partitions to the derivatives revealed interesting characteristics of the clusters (Fig E10). Velocity in cluster 5 was high for low allergen concentrations and then decreased for high doses. This implies that patients in this cluster exhibit a high response also for low doses. Cluster 1 showed a constant velocity at 0, implying no response, whereas the other clusters present low velocity for low doses that quickly increase until their highest response is reached. The moderate responses presented high variability in terms of velocity and acceleration.

#### K-means clustering on specific IgE data

To compare the discriminatory power of IgE in comparison with the MAT assay, we also ran k-means clustering on specific IgE data (Fig E11). The optimal number of clusters was 2, with the clustering distinguishing between patients with low versus those with high sIgE levels.
